# Validity and Reliability of Point-of-Care Ultrasound for Detecting Moderate- or High-Grade Carotid Atherosclerosis in an Outpatient Department

**DOI:** 10.3390/diagnostics13111952

**Published:** 2023-06-02

**Authors:** Wan-Ling Chang, Pei-Ya Chen, Po-Jen Hsu, Shinn-Kuang Lin

**Affiliations:** 1 Stroke Center and Department of Neurology, Taipei Tzu Chi Hospital, Buddhist Tzu Chi Medical Foundation, New Taipei City 23142, Taiwan; 2 School of Medicine, Tzu Chi University, Hualien 97004, Taiwan

**Keywords:** carotid atherosclerosis, carotid plaque score, carotid sonography, outpatient department, point-of-care ultrasound, risk factors

## Abstract

The prevalence of carotid stenosis is considerably higher in asymptomatic individuals with multiple risk factors than in the general population. We investigated the validity and reliability of carotid point-of-care ultrasound (POCUS) for rapid carotid atherosclerosis screening. We prospectively enrolled asymptomatic individuals with carotid risk scores of ≥7 who underwent outpatient carotid POCUS and laboratory carotid sonography. Their simplified carotid plaque scores (sCPSs) and Handa’s carotid plaque scores (hCPSs) were compared. Of 60 patients (median age, 81.9 years), 50% were diagnosed as having moderate- or high-grade carotid atherosclerosis. The overestimation and underestimation of outpatient sCPSs were more likely in patients with low and high laboratory-derived sCPSs, respectively. Bland–Altman plots indicated that the mean differences between the participants’ outpatients and laboratory sCPSs were within two standard deviations of their laboratory sCPSs. A Spearman’s rank correlation coefficient revealed a strong positive linear correlation between outpatient and laboratory sCPSs (r = 0.956, *p* < 0.001). An intraclass correlation coefficient analysis indicated excellent reliability between the two methods (0.954). Both carotid risk score and sCPS were positively and linearly correlated with laboratory hCPS. Our findings indicate that POCUS has satisfactory agreement, strong correlation, and excellent reliability with laboratory carotid sonography, making it suitable for rapid screening of carotid atherosclerosis in high-risk patients.

## 1. Introduction

Traditional risk factors for stroke include advanced age, hypertension, diabetes mellitus, hyperlipidemia, cardiac or coronary artery disease, obesity, and tobacco smoking [1]. In 2010, the American Heart Association introduced a framework for ideal cardiovascular health called Life’s Simple 7, which are seven predictors (body weight, physical activity, nonsmoking, diet, total cholesterol level, blood pressure, and fasting blood glucose level) of heart health [2]. Adherence to Life’s Simple 7 may effectively reduce primary and secondary cardiovascular risks in the general population [3]. 

The aforementioned traditional risk factors also contribute to the development of carotid atherosclerosis, which may serve as a marker of the general atherosclerotic process and is associated with cardiovascular and cerebrovascular events [4,5,6]. The prevalence of carotid plaque formation has been increasing worldwide [7]. Carotid atherosclerosis screening may help identify high-risk patients who would benefit the most from intensive medical therapy. Spence et al. reported that 63% of all asymptomatic individuals had carotid plaque progression and that these patients had twice the risk of those with stable plaque [8,9]. Carotid sonography is the most convenient, noninvasive, and reproducible tool for the evaluation of carotid atherosclerosis, and its use is recommended for all patients who have experienced an ischemic stroke or a transient ischemic attack (TIA). However, the procedure is not frequently performed on patients with the aforementioned traditional risk factors in whom no cerebrovascular event has occurred. As the prevalence of asymptomatic carotid artery stenosis is low in the general population and because this disease is associated with a relatively small proportion of all stroke cases, the US Preventive Service Task Force (USPSTF) recommends a moderate level of caution against screening asymptomatic individuals (i.e., adults without a history of stroke or a neurologic sign or symptom of a TIA) for carotid artery stenosis [10]. The European Society of Cardiology does not recommend the systematic screening of carotid artery stenosis because it does not improve disease outcomes in patients with diabetes mellitus without a history of cerebrovascular disease [11]. However, the prevalence of carotid artery stenosis is considerably higher in patients with multiple risk factors than in the general population [7]. The European Society of Cardiology and the European Atherosclerosis Society still regard carotid plaque as class IIa evidence in the risk stratification of cardiovascular disease [12]. We recently demonstrated that asymptomatic individuals with carotid risk scores of ≥7 had a 50% risk of moderate-grade or high-grade carotid atherosclerosis (MHCA), highlighting the need for comprehensive carotid sonography [13].

Point-of-care ultrasound (POCUS), the concept of an ultrasound stethoscope, is defined as ultrasonography brought to the patient and performed by the provider in real time [14]. It is rapid, cost effective, and portable; therefore, it has diverse uses in medical settings, particularly in the emergency department, with applications ranging from screening and diagnosis to procedural guidance and monitoring, and it has become crucial for shared clinical decision making [15]. However, its diagnostic accuracy varies depending on the skill of the operator, quality of the ultrasound device, location of the target organ, and time required for the procedure. Rapid carotid POCUS screening may not be necessary for symptomatic patients who require comprehensive carotid sonography. It is more valuable for use on asymptomatic individuals or individuals with vascular risk factors. Therefore, in this study, we evaluated the validity and reliability of carotid POCUS for detection of carotid atherosclerosis in asymptomatic individuals with multiple risk factors in an outpatient setting.

## 2. Materials and Methods

### 2.1. Design and Participants

This prospective study was conducted in a neurological outpatient department (OPD) between August 2022 and November 2022. This study was approved by the Institutional Review Board of Taipei Tzu Chi Hospital, Buddhist Tzu Chi Medical Foundation (approval number: 11X-040), and it was conducted in accordance with the principles of the Declaration of Helsinki. All included participants provided written informed consent. 

For each patient presenting to the OPD, we calculated the carotid risk score, which was described in our previous study evaluating the risk of MHCA [13]. The carotid score is a composite of graded scores assigned to the following seven key risk factors (Table 1): advanced age, male sex, hypertension, diabetes mellitus, hyperlipidemia, coronary artery disease (CAD), and nonvegetarian diet. Thus, the total score can range from 0 to 17 points. Each score represents a matched risk of MHCA, and a total score of ≥7 indicates a ≥50% risk of MHCA. Patients with detailed medical history records and a carotid risk score of ≥7 were included in our study. We excluded patients with a history of ischemic stroke or TIA and those who underwent carotid duplex sonography in the previous 5 years.

Any cardiologist-diagnosed ischemic heart condition was considered to indicate a history of CAD. Patients who had followed a vegetarian diet (i.e., consuming no animal products, with or without eggs) for ≥1 year were regarded as vegetarians [16,17].

### 2.2. Instruments and Measurements

**Figure 1:**
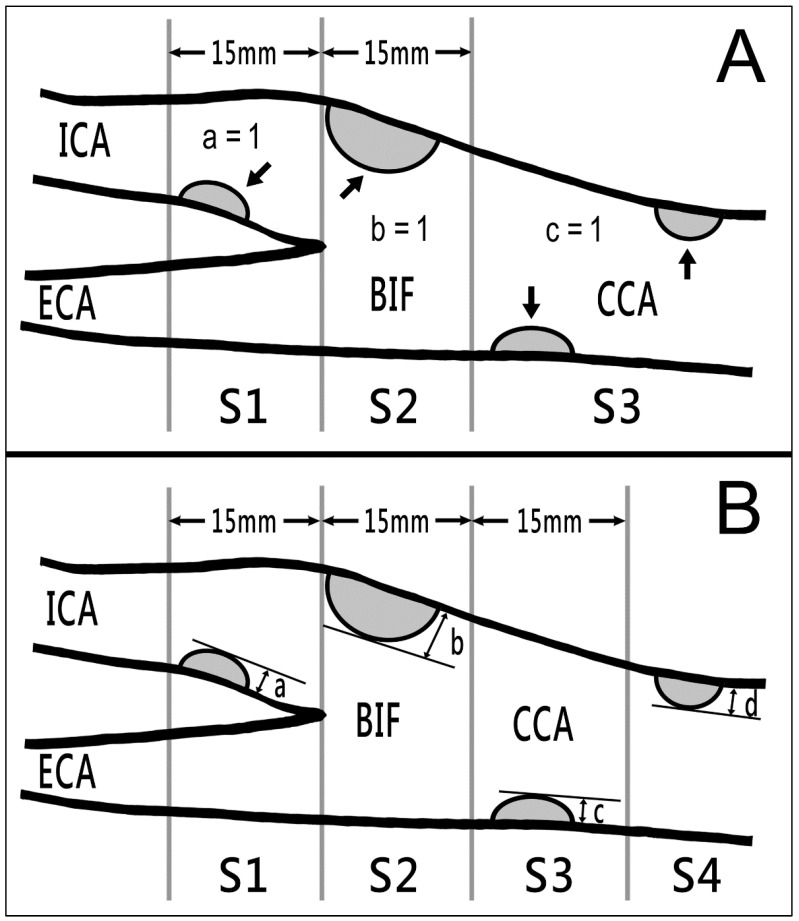
Two types of carotid plaque scores obtained using B-mode ultrasound. (**A**) sCPSs were calculated by counting the number of areas of the bilateral carotid arteries (the CCA, carotid bifurcation, and proximal ICA) with 1 or more plaques (arrows; presence = 1; absence = 0), with total scores ranging from 0 to 6. (**B**) hCPSs were computed by summing the maximal plaque thickness (in millimeters) in segments (a) S1 (region of ICA < 15 mm distal to bifurcation), (b) S2 (region of ICA and CCA < 15 mm proximal to bifurcation), (c) S3 (region of CCA 15–30 mm proximal to bifurcation), and (d) S4 (region of CCA > 30 mm proximal to bifurcation) on both sides. BIF, bifurcation; CCA, common carotid artery; ECA, external carotid artery; hCPS, Handa’s carotid plaque score; ICA, internal carotid artery; sCPS, simplified carotid plaque score.

After enrollment, the patients first underwent carotid POCUS in the OPD by an experienced stroke neurologist by using a wireless pocket-size device (ASUS Portable Ultrasound Scanner LU700L; ASUSTek Computer, Taipei, Taiwan) with a 5–10 MHz transducer combining real-time color B-mode imaging with pulsed Doppler imaging. For the procedure, the patients were seated with the chin slightly extended upward. Sonographic images were displayed on and recorded using a tablet through a Wi-Fi connection between the scanner and the tablet. The neurologist (SKL) used real-time B-mode imaging (both transverse and longitudinal sections) from the proximal common carotid artery (CCA) to the proximal internal carotid artery (ICA) on both sides. A carotid plaque was defined as local intima–media thickness that was 50% greater than that of the surrounding vessel wall or a local intima–media thickness of >1.5 mm [18,19]. In the interest of rapid screening, we did not measure plaque thickness, perform color Doppler flow imaging, or evaluate Doppler flow velocity; only visual detection was performed. Simplified carotid plaque scores (sCPSs) were calculated by counting the number of areas of the carotid artery (bilateral CCA, carotid bifurcation, and proximal ICA) with ≥1 plaques (presence of ≥1 plaques = 1, no plaque = 0; Figure 1A) [20], with the total sCPS ranging from 0 to 6. The results were recorded as outpatient sCPS. The entire procedure of outpatient carotid POCUS, from turning on the device to saving the final image file, was completed within 3 min.

The participants also underwent comprehensive carotid duplex sonography at a sonographic laboratory within 2 weeks of the POCUS screening. The procedure was performed with the patient in the supine position and included color Doppler flow imaging and the evaluation of the Doppler flow velocities and flow volumes of the bilateral carotid and vertebral arteries. Sonographers (with ≥10 years of experience) performed comprehensive carotid duplex sonography by using the Affiniti 70 Ultrasound System (Philips Healthcare, Bothell, WA, USA) with a 3–12 MHz transducer combining real-time color B-mode imaging with pulsed Doppler imaging. Another carotid plaque score as described by Handa et al. [21] (termed as Handa’s carotid plaque score (hCPS)) was calculated by summing the maximal plaque thicknesses measured on the near and far walls at each of the four segments on bilateral carotid arteries (Figure 1B). Both sCPS and hCPS were measured. The average time required for this procedure was approximately 25 min. On the basis of hCPSs, the patients were divided into mild (score, 1.1–5.0), moderate (score, 5.1–10.0), and severe (score, >10.0) atherosclerosis groups. An hCPS of >5 was defined as MHCA [22,23]. Another stroke neurologist (PJH) interpreted the findings of comprehensive carotid sonography and calculated the participants’ sCPSs and hCPSs. The results of sCPS and hCPS at the sonographic laboratory were recorded as laboratory sCPS and laboratory hCPS, respectively. We compared the outpatient and laboratory sCPSs in terms of agreement and reliability and investigated the correlations between carotid risk score, sCPS, and hCPS.

Although sCPS and hCPS assessments have been widely used for evaluation of the degree of carotid atherosclerosis, we still conducted the intraobserver and interobserver studies to assure the reliabilities of laboratory sCPS and hCPS. For the intraobserver study, a stroke neurologist (PJH) performed the measurements on the first 30 participants twice, two weeks apart. For the interobserver study, two stroke neurologists (PJH and PYC) performed the measurements of laboratory sCPS and hCPS on the same 30 participants.

### 2.3. Statistical Analyses

Due to the nonnormal distribution of the measured variables, data are presented as medians (interquartile range). The Mann–Whitney *U* test was used to compare continuous variables. *p* < 0.05 indicated statistical significance. We used the intraclass correlation coefficient (ICC), including 95% confidence interval (CI), together with a weighted kappa value to determine the intraobserver and interobserver reliabilities in the assessment of laboratory sCPS and hCPS. Given that comprehensive carotid sonography is regarded as the standard method for the detection of carotid atherosclerosis, laboratory sCPS was regarded as the standard tool for calculating the participants’ sCPSs. The Bland–Altman test, Spearman’s rank correlation coefficient, and intraclass correlation coefficient (ICC) were performed to evaluate the agreement, correlation, and reliability between outpatient and laboratory sCPSs. ICC values range from 0 to 1; a value closer to 1 indicates a higher level of homogeneity. An ICC value between 0.75 and 0.90 indicates good reliability, and an ICC value greater than 0.90 indicates excellent reliability [24]. The agreement between measurements or observers is considered strong if the weighted kappa value is above 0.80 [25]. All statistical analyses were performed using SPSS (version 24, IBM, Armonk, NY, USA), MedCalc (version 18, MedCalc Software bvbd, Ostend, Belgium), and JMP (version 17, SAS Campus Drive, Cary, NC, USA).

## 3. Results

### 3.1. Participant Characteristics

**Figure 2:**
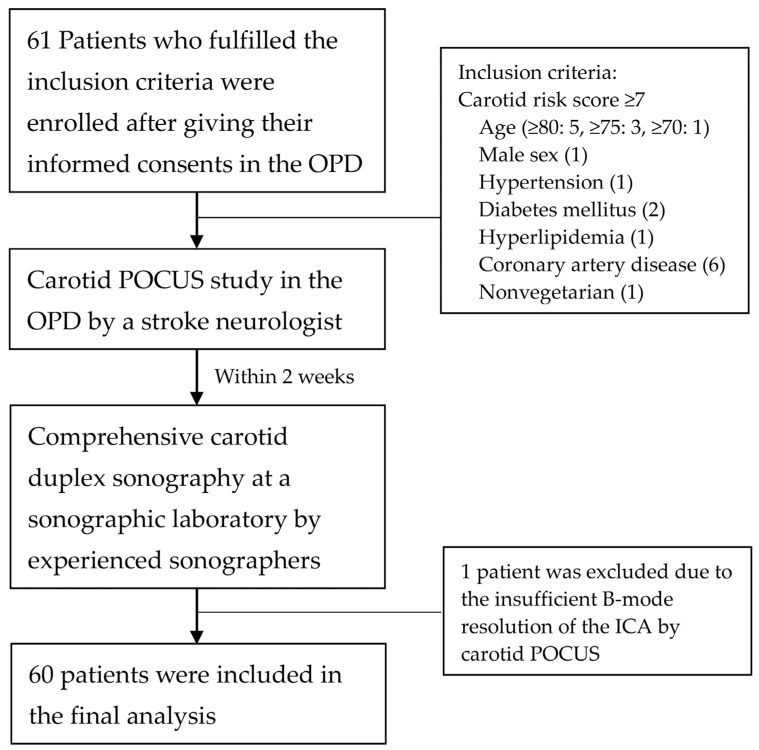
Flowchart of patient enrollment. OPD, outpatient department; POCUS, point-of-care ultrasound. ICA, internal carotid artery.

We enrolled 61 consecutive patients meeting the inclusion criteria. One patient was excluded due to insufficient B-mode resolution of the ICA because of a short neck, the relatively high location of the CCA bifurcation, and obesity, which interfered with the administration of outpatient POCUS with the patient in a sitting position. Finally, 60 patients (40 women and 20 men; median age, 81.9 [79.7–85.2] years) were included in the analysis. Figure 2 presents the patient enrollment flowchart. Table 2 summarizes the patients’ baseline clinicodemographic characteristics, including carotid risk scores. 

Approximately two-thirds of the participants presented to the OPD with a diagnosis of dementia. Other clinical syndromes included dizziness or vertigo, Parkinson’s disease, hypertension, and neuromuscular disorder. Hypertension and nonvegetarian diet were the predominant risk factors (noted in 87% of all participants), followed by hyperlipidemia, diabetes mellitus, and CAD. Only the patients with CAD (*n* = 16) received antiplatelet treatment for vascular disease. Based on the inclusion criteria, the carotid risk scores of the patients were ≥7. More than half of the patients (58%) had a carotid risk score of 7 or 8 (median: 8, highest: 17). No between-sex differences were observed in age, risk factors, or carotid risk scores.

### 3.2. Assessment Reliabilities of Laboratory sCPS and hCPS

Table 3 presents the intraobserver and interobserver reliabilities in assessment of the laboratory sCPS and hCPS on the first 30 participants. For intraoberver assessment, the ICC values of laboratory sCPS and hCPS exhibited excellent reliabities and the weighted kappa values exhibited strong agreements betweem two measurements. For interobserver assessment, the ICC values exhibited good to excellent reliabilities and the weighted kappa values also exhibited strong agreements between two observers.

### 3.3. Carotid Sonography Findings

**Figure 3:**
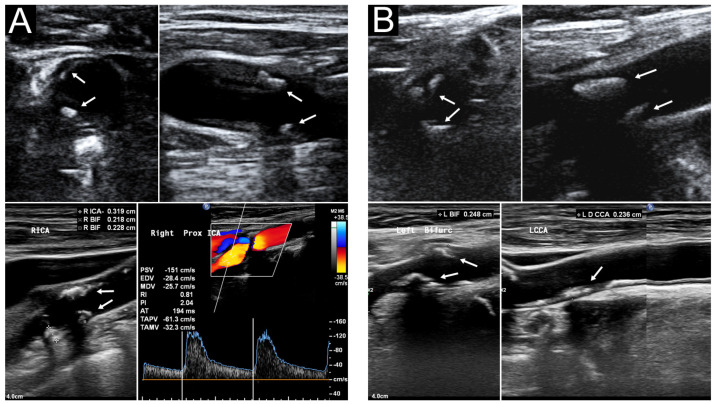
Carotid ultrasound of a 91−year−old woman. Her outpatient sCPS, laboratory sCPS, and laboratory hCPS were 6, 6, and 19.5, respectively. (**A**) Segmental heteroechogenic plaques (arrows) were detected in the right carotid bifurcation (BIF) and internal carotid artery (ICA) through transverse (upper left) and longitudinal (upper right) POCUS performed in the outpatient department. Laboratory sonography revealed the same segmental plaques more clearly with various plaque thickness (lower left). Color duplex sonography indicated an elevated peak systolic velocity in the ICA (lower right). (**B**) Segmental plaques (arrows) were detected in the left carotid BIF and ICA through transverse (upper left) and longitudinal (upper right) approach POCUS. Laboratory sonography indicated more segmental plaques extending to the common carotid artery (lower right). BIF, right carotid bifurcation; hCPS, Handa’s carotid plaque score; ICA, internal carotid artery; POCUS, point-of-care ultrasound; sCPS, simplified carotid plaque score.

Figure 3 presents the sonographic findings of carotid plaques in a 91−year−old woman with a carotid risk score of 13. Table 4 summarizes the carotid sonography results of all 60 patients. We previously [13] reported that a median carotid risk score of 8 indicates a ≥63.7% risk of MHCA. The median outpatient and laboratory-derived sCPS was 2 (range: 0–6), and the median laboratory-derived hCPS was 5.0 (range: 0–19.5). No between-sex differences were noted in the sCPSs and hCPS. Thirty patients (50%) were found to have MHCA (hCPS > 5), which is consistent with our study revealing that a carotid risk score of ≥7 confers a 50% higher risk of MHCA [13]. No differences in risk factors were observed between patients with laboratory hCPSs ≤ 5 and those with laboratory hCPSs > 5 (Table 5). Patients with laboratory hCPSs > 5 had higher outpatient and laboratory sCPSs than those with laboratory hCPSs ≤ 5. 

**Figure 4:**
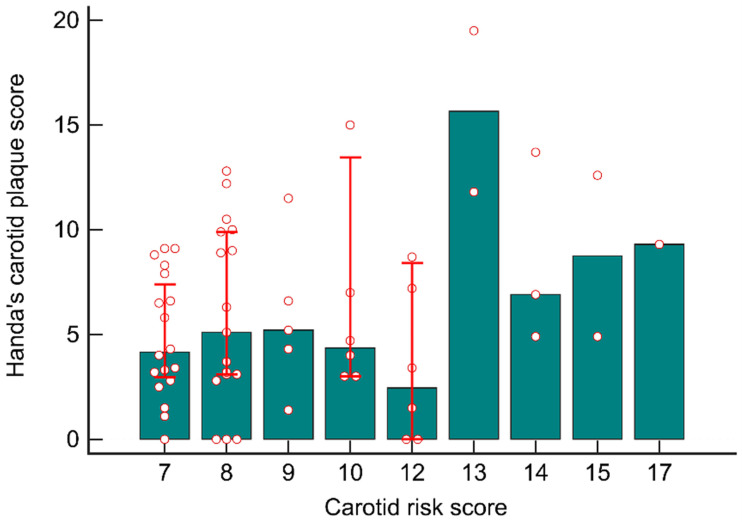
Distributions of the participants’ hCPS based on initial carotid risk scores. The patients with a carotid risk score of >12 had a higher hCPS than did those with a carotid risk score of ≤12.

The distribution of the participants’ hCPSs based on their initial carotid risk scores is presented in Figure 4. The median laboratory-derived hCPS was 4.2 (2.8–7.9) in patients with a carotid risk score of 7 (*n* = 18) and 5.1 (3.0–9.9) in those with a carotid risk score of 8 (*n* = 17). The patients with carotid risk scores of >12 (*n* = 8) had significantly higher median laboratory-derived hCPSs than did those with carotid risk scores of ≤12 (10.6 [5.9–13.2] vs. 4.3 [2.9–8.5]; *p* = 0.005).

### 3.4. Agreement and Reliability of Outpatient Carotid POCUS

**Figure 5:**
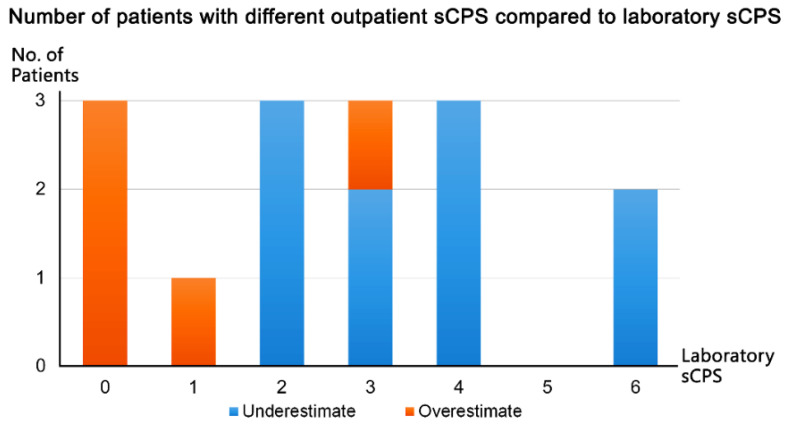
Discrepancy in implified carotid plaque score (sCPS) between the outpatient and laboratory measurements.

We compared the participants’ outpatient sCPSs with their laboratory sCPSs. sCPS discrepancy between the outpatient and laboratory measurements was noted in 15 of 60 patients (25%), with the difference being only 1 point. These scores were overestimated in 5 patients (laboratory sCPSs of 0, 1, and 3) and underestimated in 10 (laboratory sCPSs of ≥2; Figure 5).

**Figure 6:**
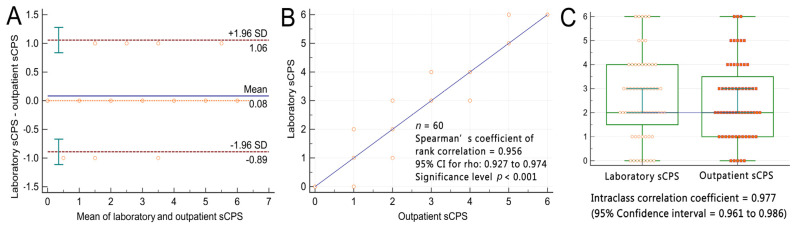
Participants’ outpatient and laboratory simplified carotid plaque scores. (**A**) Bland−Altman plots indicate that the mean differences in the participants’ outpatient and laboratory-derived sCPSs were within ±2 standard deviations of their laboratory sCPSs. The blue continuous line labeled as “mean” indicates the bias, the red dashed lines labeled as “1.96 SD” indicate the level of agreement, and the green bars indicate the 95% confidence interval. (**B**) Spearman’s rank correlation coefficient plots revealed a strong linear correlation between the outpatient and laboratory-derived sCPSs. (**C**) Intraclass correlation coefficient analysis revealed excellent reliability between the outpatient and laboratory sCPSs. sCPS, simplified carotid plaque score.

Bland–Altman plots indicated that the mean differences between the participants’ outpatient and laboratory sCPSs were within ±2 standard deviations (i.e., within a 95% limit of agreement) of their laboratory-derived sCPSs (Figure 6A). The bias (mean difference) between the two methods was only 0.08 points. Spearman’s rank correlation coefficient analysis revealed a strong positive, linear correlation between the two methods (r = 0.956; *p* < 0.001; Figure 6B). A two-way mixed-effects model with absolute agreement ICC analysis indicated excellent reliability between the two methods (ICC = 0.954, 95% confidence interval: 0.925–0.972; Figure 6C). A power exploration for the equivalence between outpatient and laboratory sCPSs using JMP software with a sample size of 60 per group yielded a statistical power of 0.889. This indicates an 88.9% likelihood of detecting a significant difference of 0.083 in sCPS at a significance level of 0.05, with a total sample size of 120 measurements.

Table 6 presents the linear correlations between the participants’ carotid risk scores, outpatient and laboratory sCPSs, and laboratory hCPSs. Carotid risk scores were positively and linearly correlated with both outpatient and laboratory sCPSs and laboratory hCPS. Both outpatient and laboratory sCPSs were positively and linearly correlated with laboratory hCPS. 

## 4. Discussion

We demonstrated the validity of outpatient POCUS for the rapid (≤3 min) screening of carotid atherosclerosis in high-risk patients. In our cohort, 50% of the patients with carotid risk scores of ≥7 were diagnosed as having MHCA (laboratory hCPS > 5). sCPSs were more likely to be overestimated (underestimated) in patients with low (high) laboratory sCPSs. The participants’ outpatient sCPSs exhibited satisfactory agreement, strong correlations, and excellent reliability with their laboratory sCPSs. Higher carotid risk scores indicate higher sCPSs and hCPSs.

Plaque scores, rather than plaque morphology, are widely used to predict plaque burden and vascular risk. Various scoring systems with satisfactory predictive ability have been developed for the quantitative assessment of carotid plaques [21,26,27,28,29,30]. We previously evaluated the hCPSs of the respondents of our community survey because of the convenience of calculation; this score can be calculated by simply summing the maximal plaque thicknesses measured on the near and far walls at each of the four areas on both sides of the carotid arteries without consideration of plaque morphology [13]. In the present study, we selected sCPS for outpatient POCUS because it is a convenient, time-saving, and semiquantitative approach, with no requirement for measuring plaque thickness. The average time required for the calculation of sCPS through outpatient carotid POCUS may be shortened to <3 min if the assessment is performed with the patient in a suitable sitting position; in this position, the patient does not need to change posture except to turn the body to an appropriate angle to facilitate the detection of the bilateral carotid arteries. The wireless ultrasound device used enables physicians to move the scanner freely without any limitation related to distance from the tablet. Thus, physicians can perform rapid bedside carotid POCUS screening as part of a physical examination, similar to the stethoscope-based examination of the patient’s heart and breathing sounds.

The overestimation of outpatient sCPSs was more likely in patients with lower laboratory sCPSs. This might be because the apparent (visual) local thickening (<50%) of the intima–media layer may be regarded as a carotid plaque alongside a relatively smooth and normal carotid wall. The underestimation of outpatient sCPSs was more likely in patients with higher laboratory-derived sCPSs. Segmental plaques across the CCA bifurcation and ICA were categorized into one area on rapid POCUS but two areas on laboratory comprehensive sonography, in which the landmark of the vessel walls could be clearly visualized without any time limit, with a score of 1 and 2, respectively. However, this discrepancy in scores did not affect the consistency of the evaluation results.

We demonstrated satisfactory agreement and a strongly positive linear correlation (r = 0.956) between outpatient and laboratory sCPSs. An ICC of 0.954 with 95% confidence interval of 0.925–0.972 between the outpatient and laboratory sCPS measurements suggests considerably high similarity between the measurement results, which indicated that the measurements had excellent reliability (ICC > 0.90) [24]. We further found significantly positive linear correlations between outpatient and laboratory-derived sCPSs and laboratory-derived hCPSs. The calculation of hCPS is more time consuming than that of sCPS; nevertheless, hCPS offers a higher level of detail and a wider range of plaque scores to better reflect atherosclerosis compared with sCPS. These findings suggest that sCPS is a suitable plaque score for the POCUS-based screening of carotid atherosclerosis in the OPD.

Certain hypoechoic plaques may be overlooked when real-time B-mode carotid ultrasound is performed without color Doppler flow imaging, but the detection rate can be increased with improved pixel resolution of real-time B-mode imaging. Jang et al. reported that 39 of 801 (5%) asymptomatic individuals had hypoechoic plaque [23]. Furthermore, Polak et al. observed that 856 of 4886 (17.5%) asymptomatic individuals aged ≥ 65 years had hypoechoic plaques without any visible echogenic cap in the ICA [31]. Among them, 30 individuals (0.6%) had a stroke within 3.3 years. Only a small portion of ICA hypoechoic plaques without any visible echogenic cap escape detection; nonetheless, this is problematic because hypoechoic plaques are associated with vascular events and thus regarded as high-risk plaques. Currently, most newly developed pocket-size POCUS devices facilitate high-resolution real-time B-mode imaging with color Doppler flow imaging. Another unusual scenario is distal ICA stenosis or occlusion without any apparent plaque within the examined ICA range; this condition can only be diagnosed on the basis of increased resistance and reduced blood flow volume noted during Doppler flow imaging. However, Yang et al. [32] reported that the incidence rate of the aforementioned condition was considerably low, accounting for only 0.5% of all laboratory carotid sonography assessments.

Although no special medications are prescribed for mild- or moderate-grade carotid atherosclerosis, patients’ awareness of their subclinical carotid atherosclerosis may help reduce their cardiovascular risk, possibly by enhancing their compliance with medications and encouraging them to implement healthy lifestyle changes [33]. Asymptomatic individuals with MHCA require intensive therapy. Spence et al. formulated a new paradigm of “treating arteries instead of treating risk factors”. They observed that more intensive treatment of plaque progression resulted in a successful reversed portion of plaque progression versus regression in asymptomatic patients with carotid stenosis, considerably decreasing the incidence of cardiovascular events [34]. They emphasized that treating arteries without examining plaques is comparable to treating hypertension without measuring blood pressure.

The USPSTF reported that the harms of screening asymptomatic individuals for carotid artery stenosis outweigh the benefits [10]. Extensive laboratory-based comprehensive carotid sonography for asymptomatic individuals with one or more risk factors is not recommended because of the low incidence of carotid atherosclerosis and low cost-effectiveness of this procedure. Nevertheless, the noninvasive screening of subclinical atherosclerosis should be implemented to promote early diagnosis in high-risk patients [10]. In a study including 5808 adults from the United States, Baber et al. observed that the risks of major adverse cardiac events increased with carotid plaque burden and coronary artery calcification [5]. They recommended further cost-effective analyses to define the optimal roles of these complementary noninvasive techniques. We previously indicated the requirement for comprehensive carotid sonography in patients with carotid risk scores of ≥7, which indicates a >50% risk of MHCA. In the present study, we further recommend outpatient POCUS-based sCPS calculation for the screening of patients with multiple risk factors, which might be extended to patients with a lower carotid risk score, depending on clinical judgment. Outpatient POCUS can facilitate the rapid and cost-effective screening of carotid burden in patients with one or more risk factors. This tool may help avoid unnecessary comprehensive carotid ultrasonography in patients with carotid risk scores of ≥7 and considerably low sCPSs and also help identify patients with carotid risk scores of <7 but and high sCPSs. At present, POCUS devices are not widely used. The development of wireless, high-resolution, and cost-effective pocket-size ultrasound devices opens up new avenues for future prospective studies on the advantages and novel applications of outpatient carotid POCUS for patients with various risk factors.

This study has some limitations. First, a skilled and experience clinician is required to perform outpatient carotid POCUS with the patient in a sitting position, particularly if the location of CCA bifurcation is relatively high. Second, we did not perform intrarater and interrater reliability tests. Ultrasound is a highly operator-dependent procedure; therefore, sonographers must receive rigorous training. Richter et al. reported excellent interrater agreement (experienced and certified vascular neurologists) and satisfactory inter method agreement (compared with angiography) for the sonographic grading of extracranial ICA stenosis [35]. Andersen et al. focused on outpatient cardiac auscultation performed for the detection of cardiac murmur by using a stethoscope; they reported that both intrarater agreement and agreement with the reference considerably varied among general practitioners, cardiologists, and medical students [36]. They found that >5 years of experience in clinical practice and cardiology specialty was strongly associated with agreement between the obtained and reference results of cardiac murmur. In the study of Suttie et al., a trained medical student, an emergency department resident, and emergency department physicians used POCUS to detect carotid artery stenosis in patients with acute stroke or TIA presenting to the emergency department [37]. The authors reported that the results of carotid POCUS had a low to moderate association with those of computed tomography angiography for detecting ≥ 50% stenosis. Similarly, Saxhaug et al. conducted a carotid POCUS study in the ward, wherein experienced cardiologists evaluated patients hospitalized because of acute stroke or TIA for carotid artery stenosis [38]. Their study indicated satisfactory agreement between the handheld ultrasound device and conventional duplex ultrasound or computed tomography angiography for the classification of carotid stenosis. In the present study, carotid POCUS was performed by a stroke neurologist specializing in neurosonology and with >30 years of clinical experience. Laboratory carotid sonography was performed by experienced sonographers with ≥10 years of relevant experience, and the results were interpreted by another stroke neurologist with ≥5 years of relevant experience. Sufficient training is crucial for quality to be maintained. Nevertheless, studies could further examine the interrater agreement studies of carotid POCUS to help clarify the benefits of its clinical application. Third, as previously mentioned, hypoechoic plaque or distal ICA stenosis or occlusion is likely to be overlooked during real-time B-mode imaging. To save time, we performed only the visual assessment of plaques for calculating the participants’ outpatient sCPSs even though color Doppler flow imaging and Doppler flow detection are built-in functions of the POCUS device used in this study. Laboratory hCPSs can be accurately determined through the measurement of plaque thickness, but this process is time consuming. Nevertheless, the correlation we observed between sCPS and hCPS was strong.

## 5. Conclusions

Wireless carotid POCUS is a suitable tool for the rapid screening of MHCA in high-risk patients in the OPD. The results of POCUS exhibited satisfactory agreement, strong correlation, and excellent reliability with those of comprehensive laboratory-based carotid sonography. The extensive implementation of carotid POCUS screening in the OPD can help increase the detection rate of subclinical carotid atherosclerosis in patients with various risk factors.

## Figures and Tables

**Table 1:** Carotid risk score components.

Items	Score
Age	
<70 years	0
70–74 years	1
75–79 years	3
≥80 years	5
Male sex	1
Hypertension	1
Diabetes mellitus	2
Hyperlipidemia	1
Coronary artery disease	6
Nonvegetarian	1
Total score	0–17

A carotid risk score of ≥7 indicates a ≥50% probability of a moderate or high degree of carotid atherosclerosis (sensitivity: 100%, specificity: 98%, area under the receiver operating characteristic curve: 0.977).

**Table 2:** Basic clinicodemographic characteristics of the participants.

Characteristics	Total (*n* = 60)	Male (*n* = 20)	Female (*n* = 40)	*p* Value
Clinical features				0.670
Dementia	39 (65.1%)	13 (65%)	26 (65%)	
Dizziness/vertigo	6 (10.0%)	2 (10%)	4 (10%)	
Parkinson’s disease	5 (8.3%)	1 (5%)	4 (10%)	
Hypertension	5 (8.3%)	1 (5%)	4 (10%)	
Neuromuscular disorder	5 (8.3%)	3 (15%)	2 (5%)	
Risk factors				
Age	81.9 (79.7–85.2)	81.5 (77.9–84.2)	82.0 (80.3–87.0)	0.233
≥90 years	7 (11.7%)	1 (5%)	6 (15%)	
85–89 years	9 (15.02%)	2 (10%)	7 (18%)	
80–84 years	29 (48.3%)	11 (55%)	18 (45%)	
75–79 years	12 (20.0%)	3 (15%)	9 (22%)	
70–74 years	2 (3.3%)	2 (10%)	0 (0%)	
<70 years	1 (1.7%)	1 (5%)	0 (0%)	
Male sex	20 (33%)	-	-	-
Hypertension	52 (87%)	17 (85%)	35 (88%)	>0.999
Diabetes mellitus	20 (33%)	5 (25%)	15 (38%)	0.395
Hyperlipidemia	31 (52%)	9 (45%)	22 (55%)	0.586
Coronary artery disease	16 (27%)	7 (35%)	9 (23%)	0.359
Nonvegetarian	52 (87%)	18 (90%)	34 (85%)	0.707
Carotid risk score	8.0 (7.0–10.0)	8.0 (7.0–13.0)	8.0 (7.0–10.0)	0.519
7–8	35 (58.3%)	11 (55%)	24 (60%)	
9–12	17 (28.3%)	4 (20%)	13 (33%)	
>12	8 (13.3%)	5 (25%)	3 (7%)	

**Table 3:** Results of the intraobserver and interobserver assessments of laboratory sCPS and hCPS on 30 participants.

	Intraobserver		Interobserver	
Assessment	ICC (95% CI)	Weighted Kappa (95% CI)	ICC (95% CI)	Weighted Kappa (95% CI)
sCPS	0.958 (0.914 to 0.979)	0.934 (0.842 to 1.000)	0.939 (0.877 to 0.970)	0.844 (0.743 to 0.945)
hCPS	0.974 (0.946 to 0.987)	0.897 (0.823 to 0.972)	0.970 (0.939 to 0.986)	0.830 (0.753 to 0.907)

CI, confidence interval; ICC, intraclass correlation coefficient; hCPS, Handa’s carotid plaque score; sCPS, simplified carotid plaque score.

**Table 4:** sCPS and hCPS of all included participants.

	Total (*n* = 60)	Male (*n* = 20)	Female (*n* = 40)	*p* Value
Carotid risk score	8.0 (7.0–10.0)	8.0 (7.0–13.0)	8.0 (7.0–10.0)	0.519
Carotid risk probability (%)	63.7 (50.5–75.8)	63.7 (58.6–87.9)	61.2 (50.5–75.8)	0.137
Outpatient sCPS	2.0 (1.0–3.5)	3.0 (1.5–3.0)	2.0 (1.0–4.0)	0.719
Laboratory sCPS	2.0 (1.5–4.0)	3.0 (1.5–4.1)	2.0 (1.5–4.0)	0.555
Laboratory hCPS	5.0 (3.1–9.0)	5.9 (2.9–8.9)	4.3 (3.1–9.0)	0.742

hCPS, Handa’s carotid plaque score; sCPS, simplified carotid plaque score.

**Table 5:** Risk factors and carotid plaque scores of patients with moderate- or high-grade carotid atherosclerosis (Handa’s carotid plaque score > 5) and those without it.

	hCPS ≤ 5 (*n* = 30)	hCPS > 5 (*n* = 30)	*p* Value
Carotid risk score	8.0 (7.0–10.0)	8.0 (7.0–12.0)	0.529
Carotid risk probability (%)	58.6 (50.5–75.8)	63.7 (58.6–88.9)	0.221
sCPS at outpatient clinic	1.0 (1.0–2.0)	3.5 (3.0–5.0)	<0.001
sCPS at laboratory	1.5 (0.0–2.0)	4.0 (3.0–5.0)	<0.001
hCPS at laboratory	3.1 (1.4–3.7)	9.0 (6.9–11.5)	<0.001
Age	81.9 (80.1–87.7)	81.9 (78.7–84.4)	0.506
Male sex	8 (27%)	12 (40%)	0.412
Hypertension	26 (87%)	26 (87%)	>0.999
Diabetes mellitus	7 (23%)	13 (43%)	0.170
Hyperlipidemia	13 (43%)	18 (60%)	0.302
Coronary artery disease	8 (27%)	8 (27%)	>0.999
Nonvegetarian diet	25 (83%)	27 (90%)	0.707

hCPS, Handa’s carotid plaque score; sCPS, simplified carotid plaque score.

**Table 6:** Linear correlations between carotid risk score, sCPs, and hCPS.

	Carotid Risk Score			Outpatient sCPS			Laboratory sCPS		
	Coefficient	R^2^	*p*	Coefficient	R^2^	*p*	Coefficient	R^2^	*p*
Outpatient sCPS	0.223	0.130	0.005	-	-	-	-	-	-
Laboratory sCPS	0.215	0.104	0.012	1.03	0.916	<0.001	-	-	-
Laboratory hCPS	0.438	0.070	0.042	2.449	0.833	<0.001	2.314	0.861	<0.001

sCPS, simplified carotid plaque score; hCPS, Handa’s carotid plaque score.

## Data Availability

The data presented in this study are available on request from the corresponding author.
